# The global epidemiology of carbapenem-resistant *Acinetobacter baumannii*

**DOI:** 10.1093/jacamr/dlaf134

**Published:** 2025-07-29

**Authors:** Angelique Boutzoukas, Yohei Doi

**Affiliations:** Department of Pediatrics, Duke University, Durham, NC, USA; Duke Clinical Research Institute, Duke University, Durham, NC, USA; Division of Infectious Diseases, University of Pittsburgh School of Medicine, Pittsburgh, PA, USA; Departments of Microbiology and Infectious Diseases, Fujita Health University School of Medicine, Aichi, Japan

## Abstract

Carbapenem-resistant *Acinetobacter baumannii* (CRAb) is a challenging, environmentally hardy organism with a propensity to spread within hospitals and a predilection to infect critically ill, vulnerable patients. With its potential for rapid transmission, limited treatment options, and substantial mortality, CRAb is recognized as a critical, top-priority pathogen. Since its initial discovery in 1985, CRAb has disseminated globally, presenting a significant public health threat. CRAb is now endemic in many regions in Europe, South America, Asia, and Africa and globally contributes to over 50 000 deaths each year. Its ability to adhere to hospital surfaces, withstand desiccation, and form biofilms leads to widespread outbreaks. At-risk populations include those hospitalized and ventilated, and the most frequent presentations are respiratory and bloodstream infections. Carbapenem resistance in CRAb is primarily mediated by plasmid-borne carbapenemase genes, especially *bla*_OXA-23_. These genes, carried by several epidemic international clones, including IC1 and IC2, have facilitated the global dissemination of CRAb through horizontal gene transfer in healthcare settings. Mortality rates are >20% and vary substantially by region and by type of infection, with bloodstream infections carrying >40% mortality. Despite its significant impact, the development of treatments for CRAb remains inadequate. The novel agent sulbactam-durlobactam holds promise for improved patient outcomes, but ongoing therapeutic development, infection prevention, and antimicrobial stewardship are critical to combat this formidable pathogen. Here, we review the emergence and dissemination of CRAb, its molecular epidemiology and resistance mechanisms, summarize contemporary global clinical epidemiology and patient outcomes, and briefly describe existing and future therapeutics.

## Introduction

*Acinetobacter baumannii* is a challenging Gram-negative bacterium known for causing hard-to-treat infections primarily in healthcare settings. Unlike other *Acinetobacter* species that may be present in the environment and on human skin, *A. baumannii* identified in clinical cultures is in most cases pathogenic. Initially documented to cause ventilator-associated pneumonia in hospitalized patients,^1^  *A. baumannii* has since emerged as a formidable challenge in hospital settings capable of causing a wide range of infections, including respiratory, bloodstream, wound, burn, urinary tract infections, and meningitis.^2^ Hospitalized, critically ill patients are most susceptible to infection with *A. baumannii*. Complicating its predilection for vulnerable patients is its inherent ability to attach and adhere to surfaces in the hospital environment, resist desiccation, and form biofilms.^3^ Transmission within healthcare settings has resulted in epidemic outbreaks. Worse yet, *A. baumannii* has a distinct propensity to acquire multiple antimicrobial resistance (AMR) mechanisms. Because of its multidrug resistance, *A. baumannii* was historically treated with ‘last line’ antimicrobials such as carbapenems.

Unfortunately, many *A. baumannii* isolates have acquired resistance to carbapenems, first documented in 1985 in a patient at the Edinburgh Royal Infirmary with resistance to imipenem, the year the antibiotic became available for clinical use; today, carbapenem-resistant *A. baumannii* (CRAb) has spread worldwide and is a critical threat to global public health.^4^ CRAb infections carry with them a significant burden on healthcare systems, including prolonged hospital stays, increased mortality, increased rates of ICU admission and hospital readmission, all of which add substantial costs to already strained systems.^5,6^ Treatment options for CRAb are exceptionally limited and often associated with toxicities. Accordingly, the emergence of CRAb was associated with a strikingly high mortality rate up to 70%, compared to 25% mortality from susceptible *A. baumannii* strains among infected patients.^7^ In the 2022 AMR report, carbapenem resistance in *A. baumannii* was among the top five pathogen-drug combinations for deaths attributable to AMR, with an estimated 57 000 deaths worldwide in 2019.^8^ Despite its global burden of disease, the development of therapeutics for CRAb is severely lacking. Given its potential for spread, implications on a uniquely vulnerable population, and the high associated mortality, CRAb has been identified as a priority-1 critical pathogen by the WHO and an Urgent Threat in the U.S. CDC’s 2019 threats report.^9,10^

In this review, we summarize epidemiologic data with a focus on the last decade (2014–2024) including global and regional clinical and molecular epidemiology, outline key risk factors and clinical outcomes, and briefly discuss current and emerging treatment options for CRAb.

## History and global emergence of carbapenem-resistant *A. baumannii*

The success of *A. baumannii* as a pathogen is closely related to its ability to both upregulate intrinsic resistance mechanisms and acquire new ones. Literature from the 1970s on describes multiple resistance mechanisms at play producing the phenotypic resistance patterns observed.^11^ Inherent resistance mechanisms such as the chromosomally encoded AmpC cephalosporinases, now commonly referred to as *Acinetobacter*-derived cephalosporinase (ADC) enzymes, resulted in cephalosporin resistance; however, carbapenems had remained stable to these enzymes. Accordingly, carbapenems became the treatment of choice for *A. baumannii* infections. Under such selective pressure, *A. baumannii* developed resistance to carbapenems. Carbapenem resistance in *A. baumannii* is most commonly conferred by β-lactamases with carbapenemase activity. The first CRAb isolate identified in Scottland in 1985 had acquired *bla*_OXA-23_ (previously *bla*_ARI-1_) via a transferable plasmid, with subsequent global spread.^4^ Plasmid-borne genes are particularly successful at horizontal transfer within healthcare settings. While *bla*_OXA-23_ is the predominant acquired carbapenemase gene in *A. baumannii* globally, *bla*_OXA-40_ (also referred to as *bla*_OXA-24_) and *bla*_OXA-58_, and less commonly *bla*_OXA-143_ and *bla*_OXA-235_, have also been reported as distinct groups of acquired OXA carbapenemase genes. Further evidence demonstrated that naturally occurring, chromosomally encoded *bla*_OXA-51-like_ genes are present in most *A. baumannii* isolates and may contribute to carbapenem resistance. The chromosomally encoded *bla*_ADC-group_ cephalosporinase and *bla*_OXA-51-like_ carbapenemase genes confer the respective resistance phenotypes when their expression is increased by the presence of a strong promoter provided by insertion sequence IS*Aba1* upstream.^6,12,13^

Over the decade following the initial identification of CRAb, increasing reports described outbreaks within hospital settings from many regions of the world, oftentimes triggered by increased use of carbapenems to treat other cephalosporin-resistant infections.^14–17^ In addition, CRAb has been reported in association with war and natural disasters. During the war in Afghanistan and Iraq, outbreaks of wound infection by CRAb among injured military personnel were traced to widespread environmental contamination in field hospitals.^18^ CRAb infections have also been reported among tsunami and terrorist bombing victims with skin and soft tissue infections.^19,20^

## Molecular epidemiology

First recognized in 1954 by Brisou and Prevot, the genus *Acinetobacter* has since undergone numerous modifications as molecular and genetic identification methods improve and new species are discovered.^21^ In 2024, there are over 80 recognized species of *Acinetobacter*. Still, species identification at most laboratories is complicated, and for this reason several clinically relevant and closely genetically related *Acinetobacter* species are included in the *Acinetobacter calcoaceticus-Acinetobacter baumannii* complex (Acb). The Acb complex historically included *A. baumannii*, which accounts for the vast majority of human infections, as well as *Acinetobacter nosocomialis*, *Acinetobacter pittii*, and the environmental organism *Acinetobacter calcoaceticus.* More recently, genetically similar species, including *Acinetobacter lactucae* and *Acinetobacter seifertii,* have been included in the Acb complex.^22,23^ Readers are referred to a definitive review on the epidemiology and mechanisms of resistance in the Acb complex.^24^ Beyond identifying the Acb complex, genospecies identification, though traditionally challenging, is clinically important given the differing pathogenic characteristics and associated mortality rates across species.^25,26^  *A. baumannii* bacteraemia, for instance, is associated with over threefold increase in the odds of mortality compared to *A. pittii* or *A. nosocomialis*.^27^Nosocomial pneumonia due to *A. baumannii* and *A. nosocomialis* also presents as two distinct entities: patients infected with *A. baumannii* were more acutely ill, had more frequent lobar consolidation, and experienced a higher 14-day mortality rate (37%) compared to those infected with *A. nosocomialis* (14-day mortality: 15%).^28^ Moreover, *A. baumannii* is linked to higher rates of multidrug resistance;^28^ suggesting that identification at the complex level likely underestimates the true burden of resistance. Beyond prognostication, species-level identification is critical for guiding appropriate treatment decisions. While empirical therapies often target the Acb complex broadly, the greater propensity of *A. baumannii* for multidrug resistance necessitates more aggressive or broader-spectrum empirical coverage than typically required for *A. pittii* or *A. nosocomialis*. When laboratory identification stops at the complex level without species differentiation, clinicians risk either unnecessarily overtreating less resistant strains or undertreating more virulent ones like *A. baumannii*. Thus, improved diagnostic methods to reliably differentiate among Acb complex species are increasingly important for optimizing antimicrobial stewardship and patient outcomes.

Within *A. baumannii*, there are historically a number of lineages that have been associated with multidrug resistance, including resistance to carbapenems. The global population of *A. baumannii* clinical isolates is presently led by several epidemic clones, particularly the international clones (IC) 1 and 2, which are also referred to as global clones 1 and 2. Contemporary surveillance studies suggest that IC2 predominates in most regions other than Latin America.^29^ Other globally common clones include IC6, IC7 and IC8. IC4 and IC5 are frequently acknowledged as significant epidemic lineages in Latin America, which has distinct epidemiology.^30^ More recently reported clones include IC9 and IC11. IC3, which was previously prominent along with IC1 and IC2, is now found only infrequently.

Advances in genetic tools have allowed for richer understanding of the global epidemiology of *A. baumannii*. Initial identification of clonal Groups I and II were achieved using amplified fragment length polymorphism (AFLP) in 1996.^31^ However, AFLP was technically challenging and exhibited low reproducibility. Later, combined use of AFLP and pulsed-field gel electrophoresis (PFGE) enabled the identification of the so-called European Clones I, II and III.^32^ Subsequent studies showed these clones were more appropriately designated as international clones (ICs) given their widespread distribution. While PFGE was considered the gold standard for outbreak investigations prior to the advent of whole genome sequencing (WGS), it was less suited for broader epidemiologic studies.

Multi-locus sequence typing (MLST) was introduced for *A. baumannii* in 2005 and further applied in 2010 to define its population structure.^33,34^ MLST confirmed the presence of three clonal complexes and showed that multidrug-resistant strains clustered closely with the recognized ICs. Two MLST schemes were proposed: the ‘Oxford’ scheme and the ‘Pasteur’ scheme, each based on seven housekeeping genes and offering distinct advantages. The Oxford scheme offers finer discriminatory power but is more affected by recombination events, making it more useful for outbreak analysis but less reliable for evolutionary studies.^34^ In contrast, the Pasteur scheme, while offering slightly lower resolution, is more stable and thus better suited for studying long-term evolutionary trends and population structure.^33^

MLST largely replaced AFLP and PFGE for global epidemiology, though the latter continued to be used in acute outbreak settings. To address MLST’s limitations in resolving closely related strains and tracking transmission dynamics, WGS technologies were adopted. In 2017, Higgins and colleagues proposed a core genome MLST (cgMLST) scheme for *A. baumannii*, evaluating thousands of loci compared to the seven in traditional MLST.^35^ This approach confirmed the existence of at least eight ICs and allowed for accurate differentiation between hospital outbreaks that appeared indistinguishable by conventional MLST.^35^ Today, WGS combined with cgMLST is considered a highly reliable, high-resolution method for both outbreak investigation as well as for studying evolutionary trajectories and global clonal expansions of *A. baumannii*.

While molecular typing has clarified the global distribution and evolution of *A. baumannii* clones, understanding the mechanisms driving AMR, particularly carbapenem resistance, is equally important. In most CRAb isolates, carbapenem resistance is primarily conferred by the production of carbapenemase from an acquired carbapenemase gene. Since the initial discovery in 1985 as ARI-1, OXA-23 continues to be the predominant carbapenemase across regions and irrespective of ICs, accounting for 75% to 88% in recent international surveys, followed by OXA-40 (OXA-24), identified in 10% to 18%.^29,36^ This may be further augmented by overproduction of intrinsic OXA-51-like carbapenemases.^12^ NDM carbapenemases, an increasingly common cause of carbapenem resistance in Enterobacterales whose intermediate reservoir likely included *Acinetobacter* spp.,^37^ remains relatively uncommon in CRAb with a recent estimate of 5.5% of all CRAb isolates, as is KPC carbapenemase, recently estimated at only 0.05% of CRAb isolates. Most CRAb isolates also produce chromosomally encoded ADC cephalosporinases that contribute intrinsic resistance to penicillins and cephalosporins. Resistance to sulbactam, a β-lactamase inhibitor with direct activity against *Acinetobacter* PBP3, can be conferred by penicillin-binding protein (PBP) alterations and also by production of β-lactamases, including OXA-23 and TEM-1.^38–41^ The readers are referred to a recent comprehensive review of β-lactamase diversity in *A. baumannii* by Mack, *et al*.^42^

## Clinical epidemiology

### Clinical presentations of carbapenem-resistant *A. baumannii*

*A. baumannii* is a widespread nosocomial pathogen that presents with a wide range of clinical presentations, from wound and urinary tract infections (UTI) to ventilator-associated pneumonia, bacteraemia, osteomyelitis, septicaemia and meningitis. CRAb most commonly presents in ICU patients and those with indwelling devices or materials, reflecting the organism’s tendency to contaminate the healthcare environment, produce biofilm, and colonize and infect vulnerable patients. Pneumonia remains the most common source for *Acinetobacter* infections, with over half of *A. baumannii* infections presenting as pneumonia. *A. baumannii* is a common cause of nosocomial bacterial pneumonia,^43^ often associated with ventilator use. The majority of *A. baumannii* causing hospital-acquired pneumonia/ventilator-associated pneumonia (HAP/VAP) globally is multidrug-resistant *A. baumannii,* including CRAb, with a pooled global prevalence rate of 79.9% (95% CI 73.9%–85.4%) in a recent meta-analysis.^44^ While in some regions, such as the United States and Europe, CRAb remains a cause of VAP in less than one-quarter of cases, in other regions of the world, such as Southeast Asia, CRAb has emerged as one of the most frequent causes of HAP/VAP after the introduction of carbapenems in the 2000s and 2010s as a common treatment for VAP.^45^ In a prospective study of 27 ICUs in nine European countries, *A. baumannii* was one of the top five microbes responsible for VAP and was the most commonly isolated pathogen in hospitals in Greece and Turkey.^46^

Bloodstream infections (BSI) due to *A. baumannii* most often develop in critically ill patients with indwelling lines and are likely preceded by skin, mucous membrane, and respiratory colonization. Colonization, in combination with specific patient factors, predisposes to BSI with CRAb. In one study of ICU patients in South Korea, independent predictors of multidrug-resistant *A. baumannii* BSI included infection and respiratory failure at the time of ICU admission, maintaining mechanical ventilation and endotracheal tube (compared with tracheostomy), recent central venous catheter insertion, bacteraemia caused by other microorganisms after colonization by multidrug-resistant *A. baumannii*, and prior antimicrobial therapy.^47^ In comparison to other organisms that result in BSI in ICU patients, *A. baumannii* is a less frequent cause but is more often associated with acquisition in the ICU and is the organism most likely to display carbapenem resistance.^48^

Other infections, including wound infection, UTI, meningitis and osteomyelitis, often occur in specific patient populations with risk factors present. Wound infections due to CRAb commonly occur in burn patients and those with combat trauma wounds, likely reflecting field hospital contamination with *A. baumannii*.^49–53^ Total burn surface area, burn severity, as well as length of hospital stay, prior exposure to broad-spectrum antibiotics, ICU stay, and Acute Physiologic Assessment and Chronic Health Evaluation (APACHE) II score are risk factors for CRAb wound infection.^52^ UTI due to CRAb is challenging to diagnose as it often occurs in patients with indwelling catheters, making it difficult to discern infection from colonization. UTI from CRAb is overall uncommon, and the disease burden is low. On the other hand, CRAb meningitis presents mostly in patients with a history of neurosurgical interventions or head injuries and is a significant problem in parts of the world with high background prevalence of CRAb in healthcare settings.^54,55^

In addition to causing proven infections, *A. baumannii* frequently colonizes hospital environments and hospitalized patients’ respiratory tracts, skin wounds and medical devices. Distinguishing colonization from true infection is challenging, particularly in critically ill patients or those with chronic wounds or tracheostomies, where the organism may be isolated without signs of active infection. Patient-to-patient transmission is a major driver of colonization, especially during outbreak settings.^56^ Notably, colonization with extensively drug-resistant *A. baumannii* among ICU patients has been associated with increased 6-month mortality (hazard ratio: 1.75; 95% CI: 1.17–2.61), underscoring the clinical significance of even asymptomatic carriage.^57^ Surveillance definitions, such as those from the U.S. CDC’s National Healthcare Safety Network (NHSN), apply strict clinical and microbiological criteria to standardize reporting but may underestimate the true burden of *A. baumannii* infections.^58^ In practice, patients with signs of infection may not fulfil surveillance criteria yet still require antimicrobial treatment. Because misclassification risks both overtreatment of colonization and undertreatment of infection, accurate differentiation of infection and colonization remains critical to guide appropriate treatment decisions, optimize infection control measures and improve outcomes.

### Regional prevalence of CRAb and incidence of infections

**Figure 1.**
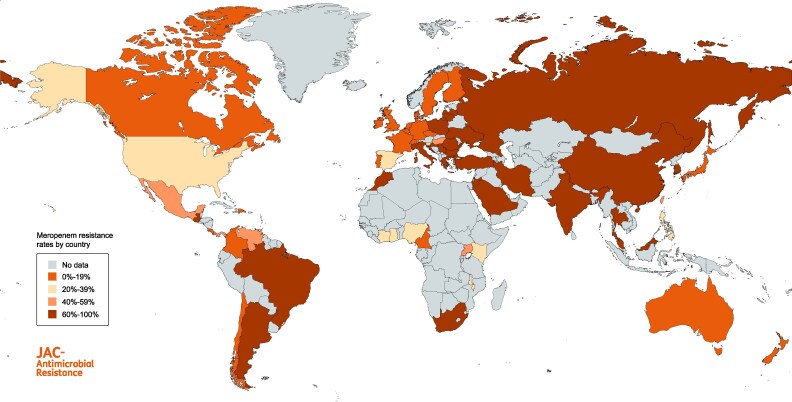
Global prevalence of meropenem resistance among *A. baumannii* isolates (2019–23, ATLAS programme). The map displays meropenem resistance rates among *A. baumannii* isolates from different countries worldwide between 2019 and 2023. Included countries and isolates were those with ≥ 10 reported isolates over 5 years. Data source: Antimicrobial Testing Leadership and Surveillance (ATLAS) programme, implemented by Pfizer.

Since its initial detection, the prevalence of CRAb has increased dramatically globally. The epidemiology of CRAb across different global regions has been outlined in recent multicentre studies and regional surveillance programmes. Prevalence of carbapenem resistance among *A. baumannii* isolates across different countries from 2019 to 2023 are shown in Figure 1, generated using data from the Antimicrobial Testing Leadership and Surveillance (ATLAS) database.^59^

Surveillance programmes such as the European Antimicrobial Resistance Surveillance Network (EARS-Net) provide annual estimates on the incidence of CRAb infections across EU countries and the European Economic Area (EEA). The 2023 estimated EU incidence of CRAb BSI was 2.98 per 100 000 population; this varies substantially between countries and regions of Europe, as do the rates of carbapenem resistance among *A. baumannii* isolates, with a range of 0%–95.8% carbapenem resistance across EU/EEA countries.^60^ Rates of carbapenem resistance follow a gradient of increasing resistance from North to South and West to East; this mirrors AMR patterns seen for other bacterial species. Northern Europe has very low rates of carbapenem resistance, with Finland and Norway reporting <1% carbapenem resistance among *A. baumannii* isolates, while countries in Southern and Southeastern Europe report ≥50% carbapenem resistance among *A. baumannii*,^60^ including Croatia, Italy, Turkey, Greece, Lithuania, Portugal and Romania, which have reported endemic spread.^61^ While both the incidence and rates of carbapenem resistance are increasing in many European countries, some countries, including Germany, Norway, Slovenia and Portugal, have reported declining rates of carbapenem resistance among hospitalized patients with *A. baumannii* from 2014 to 2018.^62,63^ Similarly, decreasing rates of carbapenem resistance from 2021 to 2023 were reported in the 2024 EARS-Net report among *A. baumannii* BSI isolates.

In the United States, the NHSN conducts national surveillance of hospital-acquired infections (HAIs). While *Acinetobacter* spp. was no longer among the top 15 overall HAI pathogens by 2021, it remained a common cause of certain infection types, including central line-associated BSIs (CLABSIs) and pneumonia and ventilator-associated pneumonia (PVAP).^64^ The epidemiology of *Acinetobacter* spp. varies by care setting: it accounts for 1% of CLABSIs in ICUs and 2% in long-term acute care hospitals (LTACHs). Among patients with PVAP, *Acinetobacter* spp. was the tenth most common pathogen in acute care hospitals and the fifth most common in LTACHs.^64^ Carbapenem resistance among *A. baumannii* HAIs is also location-dependent; in PVAP cases, 36% of isolates in acute care hospitals were carbapenem-resistant compared to 85.6% in LTACHs. While the mechanisms driving higher rates of carbapenem-resistance in LTACHs are not fully understood, both patient- and environmental-related factors are likely involved. A statewide survey in Maryland found that 100% of LTACHs sampled had patients colonized or infected with *A. baumannii*.^65^ This endemicity reflects the particularly vulnerable group of patients that reside at LTACHs, characterized by longer stays, greater comorbidity burdens, frequent healthcare exposures and common use of invasive devices such as tracheostomies and pressure wound management systems that serve as sites for acquisition of and colonization with *A. baumannii*. Moreover, frequent patient transfers between LTACHs and acute care hospitals facilitate ongoing transmission cycles. Therefore, to effectively disrupt reservoirs of CRAb and reduce transmission, LTACHs must be systematically integrated into national and international surveillance and infection control efforts.

Rates of CRAb appear to have stabilized in the USA compared to the early 2000s. While reassuring, ongoing monitoring of CRAb epidemiology remains critical to detect emerging trends and guide prevention strategies. Current surveillance systems, however, face several limitations, including underrepresentation of non-acute care settings, variable diagnostic and reporting practices across institutions, and delays in data availability. Strengthening national surveillance efforts will require enhanced standardization of reporting, broader inclusion of outpatient and long-term care facilities, and more timely data dissemination. The deployment of the U.S. CDC’s NHSN Antimicrobial Use and Resistance (AUR) Module represents a promising advancement.^66^ The AUR Module aggregates facility-level antimicrobial use and resistance data, creating an opportunity for more comprehensive and real-time monitoring of CRAb and other multidrug-resistant organisms in the coming years. Future enhancements could include direct integration with electronic health records (EHRs) through interoperable data elements, enabling seamless data transfer and facilitate calculation of infection incidence rates in addition to the more commonly reported prevalence of carbapenem resistance.

In the prospective observational Study Network of *Acinetobacter baumannii* as Carbapenem-Resistant Pathogen (SNAP) study, clinical data were collected prospectively, and whole genome sequencing was conducted in consecutive patients with clinical isolates positive for CRAb (NCT03646227) at 46 sites across 10 countries between 2017 and 2019.^36^ Of 842 patients with CRAb, approximately two-thirds acquired it in hospital, and half of patients were in an ICU at the time of positive index culture, reflecting the most vulnerable population for CRAb acquisition and infection. Approximately two-thirds of isolates caused infection rather than colonization, and the most common sources were respiratory (56% of cases), wound (20%) and bloodstream (11%). Importantly, the SNAP study did not include sites from Europe, Africa or much of Asia. A meta-analysis estimated the incidence of hospital-acquired CRAb infection across the WHO regions of Europe, Eastern Mediterranean, and Africa to be 21.4 (95% CI 11.0–41.3) cases per 1000 patients.^67^ Among patients in the ICU, the pooled incidence rate of hospital-acquired CRAb infection was 41.7 (95% CI 21.6–78.7) cases per 1000 patients; however, there was significant heterogeneity across studies. CRAb strains accounted for 13.6% (95% CI 9.7–18.7%) of all HAIs, respectively.^67^

The Central Asian and European Surveillance of Antimicrobial Resistance surveillance programme contains invasive isolates from 15 countries in Asia and non-EU countries in Europe, filling critical data gaps in areas of high resistance that are not captured in EARS-Net.^68,69^ In some global regions without international surveillance programmes, single country studies and meta-analyses of studies provide insight around CRAb epidemiology. In sub-Saharan Africa, the pooled prevalence rate of carbapenem resistance among *A. baumannii* was 20%; however, the underlying studies had high heterogeneity.^70^ Similar rates of carbapenem resistance (18.6%) were observed in a meta-analysis of studies from West African countries.^71^ Throughout Asia, single-centre and single-country studies indicate high rates of carbapenem resistance; however, there is a lack of organized surveillance across broader regions of Asia. Similar to other global regions, a broad range of carbapenem resistance rates exist, with some countries, including Japan, reporting notably low rates (<5%),^72,73^ while many nearby East Asian countries report rates exceeding 80%. In China, carbapenem resistance among *A. baumannii* clinical isolates ranged from 75%–78% during 2018–22 according to the China Antimicrobial Surveillance Network.^74^ The ATLAS programme included 2674 *A. baumannii* isolates from 13 countries in the Asia-Pacific region and found a pooled carbapenem resistance rate of 71.7% from 2012 to 2019, with the lowest rates of resistance in Japan (2.8%) and Australia (6.5%) and the highest rates of carbapenem resistance in Thailand (83%), Pakistan (85%), India (87%) and South Korea (88%).^75^ The low rates of CRAb in Japan and Australia likely reflect a combination of stringent infection control practices, predominance of *A. baumannii* lineages less prone to resistance, and a low prevalence of globally common carbapenemase genes such as *bla*_OXA-23_ and *bla*_OXA-40_, resulting in the absence of major endemic reservoirs of CRAb.^72,76^

CRAb is also highly prevalent in South America, although many isolates from this region historically belong to distinct lineages that differ from globally predominant strains (see the Molecular epidemiology section for details).^77^ Carbapenem resistance among *A. baumannii* in Latin America reaches as high as 90% in some countries.^78,79^ In Brazil, a CRAb infection rate of 0.7 per 1000 patient-days has been reported, with infections occurring most commonly among adult ICU patients, particularly those with lung infections.^80^

Additional sources of data are available to track resistance rates in countries that are typically underrepresented in global surveillance efforts. The WHO Global Antimicrobial Resistance and Use Surveillance System provides a standardized framework for countries to collect, analyse and share AMR data, incorporating laboratory, epidemiological, clinical and population-level information.^81^ The Vivli AMR Register offers another novel resource, often linked to global post-marketing studies.^82^ Both the incidence of CRAb infections and rates of AMR vary widely globally and continue to evolve, underscoring the importance of ongoing active surveillance that represents trends from all parts of the world.

### Increases in CRAb during the COVID-19 pandemic

During the COVID-19 pandemic, there was a significant increase in CRAb infections in ICUs around the world. In the United States, reports indicated a 78% rise in hospital-acquired CRAb infections from 2019 to 2020, with an estimated 7500 cases per year resulting in approximately 700 deaths.^83^ Similar trends were observed in Europe, where there was a 57% increase in BSIs caused by *Acinetobacter* spp., with carbapenem resistance rates climbing from 48.4% in 2018–19 to 65.8% in 2020–21, making *Acinetobacter* the organism with the most notable increase in BSIs of all organisms under surveillance.^84,85^ Countries with high levels of resistance prior to the pandemic saw a combined 116% increase in reported cases during this period.

The reasons for this notable rise were multifactorial. The pandemic led to a dramatic influx of patients in ICUs, overwhelming hospital capacities and straining resources. The availability of personal protective equipment (PPE), cohorting practises and other infection prevention methods were often insufficient to meet the increased demand. Additionally, diversion of resources from antimicrobial stewardship may have contributed.^86^ This combination of factors created an environment conducive to the spread of CRAb, exacerbating the incidence and resistance rates during the pandemic. In future pandemics, attention to the risk of nosocomial outbreaks caused by pathogens other than the primary pandemic agent will be essential to protect patients from adverse outcomes. Improved diagnostic stewardship and the development of rapid diagnostic tests hold significant potential to guide appropriate antibiotic use, ensuring treatment is reserved for confirmed bacterial infection. Maintaining strict adherence to infection prevention practises, particularly during periods of increased ventilator use, will be critical to minimizing the spread of CRAb and other healthcare-associated pathogens.

## Risk factors for CRAb acquisition and infection

**Figure 2.**
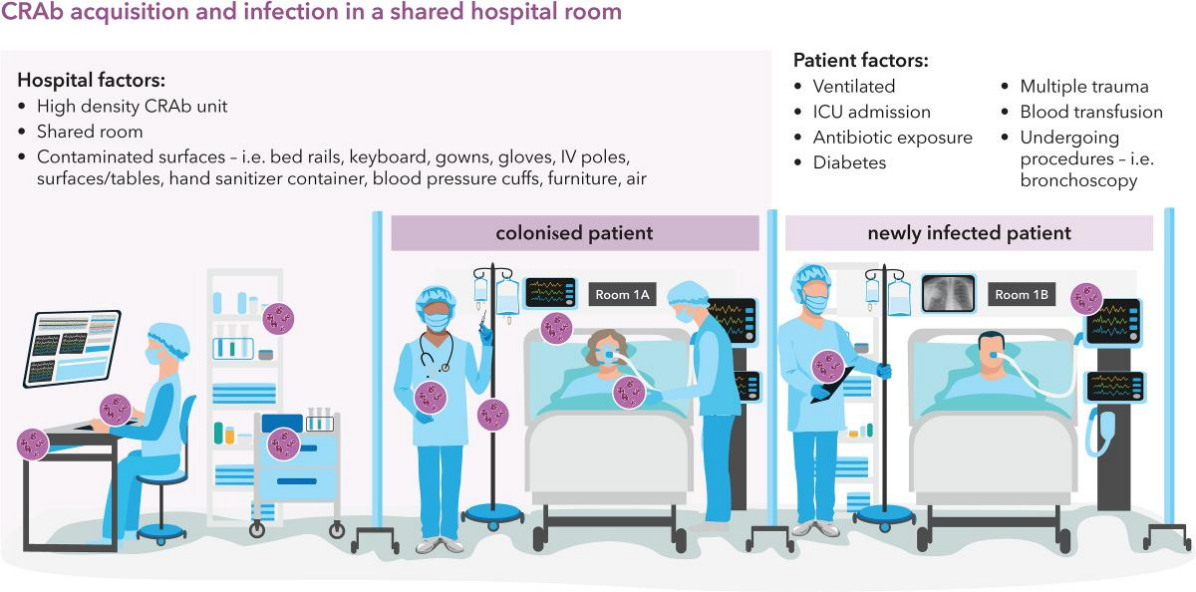
CRAb acquisition and infection in a shared hospital room. This figure depicts a shared hospital room with two patients. The patient in bed 1A is unknowingly colonized with CRAb (indicated by the infectious particles in purple), which has subsequently contaminated many surfaces of the hospital room and healthcare workers. The patient in bed 1B is now newly infected, intubated, and with hospital-acquired or ventilator-acquired pneumonia due to CRAb. The newly infected patient may have one of several patient risk factors for CRAb acquisition and/or has been exposed to one or more hospital factors that increase risk for acquisition of CRAb.

The acquisition of CRAb is frequently preceded by the interaction of various patient and environmental factors that contribute to patient susceptibility, as detailed in Figure 2. Numerous patient risk factors have been identified that are associated with the risk of CRAb acquisition during hospitalization related to degree of illness, medical conditions, medical procedures and devices, and host susceptibility. Acuity of illness and procedure-related factors that increase the risk of CRAb infection include ICU stay (with longer stay carrying greater risk), mechanical ventilation, elevated APACHE II score and invasive procedures.^87^ A history of haematologic malignancies, admission for multiple trauma, diabetes, and use of parenteral nutrition have been identified as predictors of CRAb colonization or infection.^88–90^ Finally, prior antibiotic exposures—particularly exposures to carbapenems—are independent predictors of CRAb acquisition.^87,89,90^ In ICU patients being monitored with surveillance cultures, carbapenem exposure more than quadrupled the hazard of acquiring CRAb; each additional day of exposure increased the hazard by 5%.^91^ Among patients with prior colonization with CRAb, risk factors for CRAb infection include ICU admission, cardiovascular disease, and concurrent COVID-19 infection.^92^ In patients with CRAb BSI following colonization, predictors included higher Charlson Comorbidity Index (CCI), mechanical ventilation, and multisite colonization.^93^

Environmental factors that predispose to CRAb acquisition reflect the hardy nature of *A. baumannii* and its ability to survive in the environment for prolonged periods and resist desiccation. CRAb has been detected on nearly all surfaces of ICU rooms, including bed rails, sinks, bedside tables, food tables, sheets, door handles, and even handwashing sinks.^94–96^ Devices and equipment are frequently contaminated, including IV pumps, ventilators and computer keyboards.^97,98^ In endemic hospitals, air samples have grown CRAb in up to a quarter of ICU rooms;^97^ in this setting, rectal colonization, as compared to respiratory colonization, was associated with CRAb positivity of air and environmental samples.^99^ Finally, healthcare workers may be a frequent source of contamination; CRAb has been frequently detected on the gloves, gowns, and hands of healthcare workers, underscoring the importance of hand hygiene. Exposure to a contaminated environment more than doubles the risk of acquiring CRAb (RR 2.77, 95% CI 1.50–5.13), however, this effect is thought to be modified by at least two factors: colonization pressure, or the proportion of a unit that contains patients colonized with CRAb (ICU CRAb prevalence of ≥10%), and patient location being in the trauma ICU.^100^ colonization pressure did not predict acquisition of CRAb in a burn unit in one study,^101^ but it has been shown to augment other risk factors, such that in times of high colonization pressure infection control measures become critical to prevent cross-contamination. Conversely, at times of lower colonization pressure, antimicrobial stewardship, hand hygiene, and other factors may be more important.^102^ Maintaining awareness of local colonization pressure in ICUs can allow the focus on prevention methods to shift depending on changing epidemiology.

Several studies have shown benefit from active surveillance cultures (ASC) in high-risk settings for certain organisms, such as vancomycin-resistant *Enterococcus* and methicillin-resistant *Staphylococcus aureus*, and guidelines exist to support this practice.^103^ In contrast, evidence for the impact of active surveillance of CRAb is lacking, and active surveillance of CRAb is not currently recommended by the WHO or European Society for Clinical Microbiology and Infectious Diseases (ESCMID) as critical strategies to prevent transmission of CRAb.^104,105^ Nonetheless, ASC followed by enhanced infection control after identification of CRAb have been predictive of future CRAb infections and demonstrated good specificity and negative predictive value for subsequent CRAb infection in single-centre studies, and perhaps they may be more useful in highly endemic regions or outbreak settings.^106,107^

Finally, a complex interaction exists between CRAb acquisition during acute hospitalization and subsequent discharge to LTACHs or long-term care (LTC) facilities where ongoing spread may be common. In a recent cross-section study, mechanically ventilated residents of LTC hospitals had over five times the risk of CRAb colonization as those in acute care hospitals, highlighting the importance of prevention of cross-contamination in LTC facilities in addition to LTACHs.^108^

## Diagnosis of CRAb

*Acinetobacter* species, especially those within the Acb complex, are closely related genetically, which is reflected in their similar biochemical profiles and difficulty in differentiating Acb species based on conventional biochemical testing. Species identification has improved significantly with the advent of MALDI-TOF MS;^109^ laboratories with MALDI-TOF MS instrument, such as Bruker Biotyper^®^ (Bruker Corporation, Billerica, MA, USA) and bioMérieux VITEK MS (bioMérieux SA, Marcy-l’Étoile, France) with updated databases, can typically identify *Acinetobacter* isolates at the species level.^110^ The use of MALDI-TOF MS has the added advantage of shortened turnaround time (TAT), typically by one day, over the conventional biochemical identification methods. However, antimicrobial susceptibility testing (AST) still needs to be performed by conventional methods to guide appropriate therapy, information that is not provided by MALDI-TOF MS.

To improve the TAT on AST, rapid diagnostics have been developed, and some have been approved for clinical use, using various phenotypic or genotypic approaches. VITEK^®^ REVEAL^™^ (bioMérieux SA, Marcy-l’Étoile, France) is one such platform that provides phenotypic AST results within 6 h of a positive blood culture.^111^ The current platform contains eight antimicrobial targets for *A. baumannii*, including imipenem/meropenem susceptibility. The Accelerate Pheno^™^ (Accelerate Diagnostics, Tuscon, AZ, USA) system utilizes fluorescence *in situ* hybridization to detect pathogens, identify species, and performs automated microscopic imaging to analyse bacterial growth and extrapolate minimum inhibitory concentration values.^112^ This system can identify *A. baumannii* from a positive blood culture within 2 h and provide AST results within 7 h.^113^ Notably, however, Accelerate Pheno^™^ contains targets for only piperacillin-tazobactam and amikacin, while meropenem has not been validated. While genetic platforms that detect carbapenemase genes are increasingly adopted by clinical microbiology laboratories in large hospitals, many do not include the bla_OXA_ genes most frequently harboured by CRAb. For example, the widely used BIOFIRE^®^ blood culture identification panel 2 (bioMérieux SA) contains numerous carbapenemases but notably lacks the *bla*_OXA_ genes of *A. baumannii*, including *bla*_OXA-23_. At least two lateral flow immunoassays capable of detecting OXA-23 are either cleared for *in vitro* diagnostic use or in late stages of development: The RESIST ACINETO assay (Coris BioConcept, Gembloux, Belgium) detects OXA-23, OXA-40/58 and NDM carbapenemases in *Acinetobacter* spp.,^114,115^ while the NG-Test DetectTool OXA-23 (NG-Biotech, Guipry, France) specifically targets OXA-23.^116^ Both assays enable rapid detection of these carbapenemases directly from positive blood cultures with high sensitivity and specificity, addressing a critical diagnostic gap in the rapid identification of carbapenemase production in *A. baumannii*.

Accurate species identification and rapid susceptibility results are critical to optimizing patient management and improving outcomes, but access to such technologies is still limited. In many regions, local antibiograms and carbapenem resistance rates are often relied upon to select empiric therapy, leading to overuse of broad-spectrum agents that further drives resistance development. Developing new diagnostic platforms, expanding existing ones to include more susceptibility targets, and ensuring clinical labs can access these diagnostics are crucial in combating CRAb while promoting antimicrobial stewardship.

## Clinical outcomes and their predictors

CRAb infections are associated with significant mortality, in part due to the patient factors that increase susceptibility to infection and limit treatment options. Mortality due to CRAb differs by infection type and by region, but global and regional estimates of attributable mortality due to CRAb are challenging to obtain. Most surveillance systems, including the NHSN and EARS-Net, do not capture patient outcomes. In a 2022 systematic analysis, an estimated 57 700 deaths occurred globally in 2019 that were attributable to CRAb.^8^  *A. baumannii* was the pathogen responsible for the largest portion of AMR-attributable deaths in Southeast Asia, East Asia, and Oceania. Annual deaths from CRAb are estimated in some regions of the world. The European CDC estimated 3656 deaths attributable to CRAb in 2020 in the EU and EEA.^117^

High mortality rates from CRAb infection have been observed globally, particularly in BSI or HAP/VAP, with outcomes varying by region and infection type. In the prospective, multicentre SNAP study, an overall mortality rate of 24% was reported among infected patients, compared with 14% among those classified as colonized only, highlighting the significant attributable mortality of CRAb infection. Mortality was highest among patients with bacteraemia (42%) and lowest 11% each with infection of wound and urinary tract sources.^36^ Mortality rates varied by region, with the highest rates observed in South/Central America. The most desirable outcomes among patients with CRAb occurred in China, followed by the US, with less desirable outcomes in patients in South/Central America and Australia/Singapore. A meta-analysis of studies of CRAb HAP/VAP from 29 countries demonstrated a pooled mortality associated with multidrug-resistant *A. baumannii* lung infections of 42.6% (range 28.6%–56.2%), with the highest mortality rates observed in West Asia, Southern Europe, and Northern Africa.^44^ BSIs due to CRAb are associated with particularly high mortality, often involving septic shock. In an Italian multicentre study, mortality reached 61% at 14 days and 73% at 30 days.^118^ In contrast, lower mortality rates (39%) have been reported from a single-centre study in China.^119^ The reasons for regional differences in mortality despite comparable enrollment criteria remain unclear but may involve underlying patient comorbidities, variations in healthcare delivery models, access to care, availability of supportive therapies, and treatment options. Furthermore, estimates of mortality due to CRAb are limited by variability in study design, infection definitions, and adjustment for confounders. Multicentre prospective studies with adjudicated infection status, such as the SNAP study, are robust and have the potential to enrich our understanding of attributable mortality. Future research should prioritize standardized, prospective studies with detailed severity adjustment to better define attributable mortality.

Beyond patient mortality, CRAb contributes to significant disease burden and morbidity, including increased ICU usage, prolonged hospital stays, transfer to other healthcare facilities, and new-onset chronic ventilator dependence.^5,120^ One single-centre study demonstrated 21-day ventilator dependence rates of 48.8%, highlighting the significant burden CRAb infections pose on hospitals.^121^

**Figure 3.**
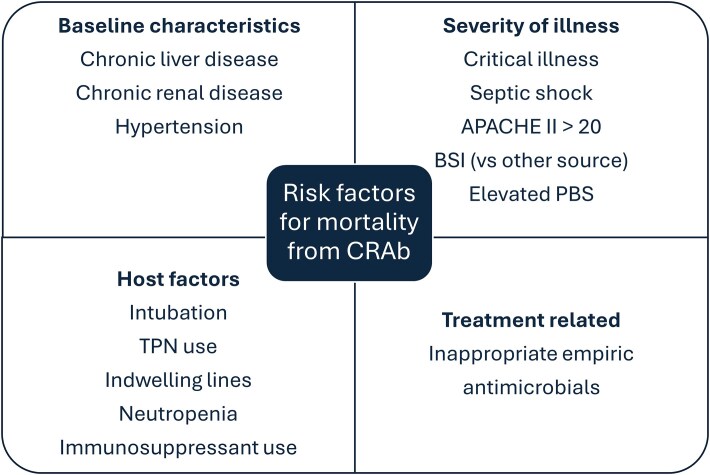
Risk factors for mortality secondary to CRAb. Patient factors that increase the risk for mortality secondary to CRAb infection are multifactorial and may be related to underlying patient conditions, severity of illness, host status, and treatment decisions. PBS, Pitt Bacteraemia Score; TPN, total parenteral nutrition.

Several predictors of mortality secondary to CRAb infections have been identified, including those related to patient conditions, severity of illness, and treatment decisions (Figure 3). Chronic liver disease, chronic renal disease, and hypertension are among the comorbidities associated with mortality from CRAb.^122^ The degree of critical illness at the time of infection is associated with mortality from CRAb, with septic shock, intubation, and elevated scores on measures of severity of illness, such as the APACHE II scores and Pitt bacteraemia scores, all shown to increase mortality risk.^122,123^ Additionally, host factors, including neutropenia and immunosuppressant use, and medical procedures and indwelling lines, including use of total parenteral nutrition, are associated with increased odds of mortality.^122^ Finally, inappropriate empiric antimicrobial prescribing is a predictor of mortality for CRAb BSI,^124^ but not for HAP/VAP source alone.^125^ In the SNAP study, independent predictors of 30-day mortality included source (BSI), monomicrobial infection and higher age-adjusted CCI score.^36^

## Treatment options

Treatment of CRAb infection is historically complex, often using multiple-drug regimens, and challenged by the toxicities as well as uncertain effectiveness of some of the available drugs. A detailed review of therapeutics currently available and in development for the treatment of CRAb is outside of the scope of this review, and we refer the reader to several recent reviews focused on treatment.^126–128^ Combination therapy is a mainstay, but the preferred combination is not defined. Combinations that include sulbactam, a β-lactamase inhibitor with intrinsic activity against *A. baumannii*, are generally the preferred backbone, and are recommended by both recommended in both Infectious Diseases Society of America (IDSA) and ESCMID guidance (Table 1).^129,130^ IDSA guidance recommends combination therapy with off-label high doses of sulbactam as an alternative regimen to sulbactam-durlobactam to optimize pharmacodynamics in the setting of sulbactam’s increasing minimum inhibitory concentrations (MICs).^130^ The potential partner agents include polymyxin B, colistin, minocycline, tigecycline, and cefiderocol and should be selected based on susceptibility testing results and specific circumstances such as site of infection and renal function. Meropenem and rifampin, though previously candidates to be combined with colistin based on synergistic activity observed in vitro, are no longer recommended after randomized clinical trials failed to show clear benefit in clinical efficacy compared with colistin alone.^131,132^

**Table 1.** Treatment guidance for CRAb from IDSA and ESCMID

	IDSA^130^	ESCMID^129^
PREFERRED REGIMEN	Sulbactam-durlobactam in combination with a carbapenem	Combination therapy including two *in vitro* active antibiotics among the available antibiotics; include ampicillin-sulbactam if active
ALTERNATIVE REGIMEN	High-dose ampicillin-sulbactam^a^ in combination with at least one other agent^b^	No recommendation^c^

IDSA, Infectious Diseases Society of America; ESCMID, European Society of Clinical Microbiology and Infectious Diseases.

^a^Total daily dose of 9 grams of the sulbactam component.

^b^Polymyxin B, minocycline > tigecycline, or cefiderocol.

^c^Recommends against polymyxin-meropenem combination, polymyxin-rifampin combination and cefiderocol.

Several phase III or IV clinical trials have been conducted in the last decade to inform the management of CRAb infections; we have summarized three late phase clinical trials in Table 2. From these trials, two new agents with CRAb activity are now available for clinical use in some regions. Sulbactam-durlobactam was approved in the U.S. in 2023 with a specific indication for the treatment of hospital-acquired bacterial pneumonia and ventilator-associated bacterial pneumonia caused by susceptible strains of *A. baumannii*. Durlobactam is a novel diazabicyclooctane β-lactamase inhibitor with a spectrum of inhibition that includes OXA carbapenemases produced by CRAb. The approval was supported by a pivotal clinical trial (ATTACK Trial) that established non-inferiority of sulbactam-durlobactam over colistin in the presence of imipenem as the background therapy for the treatment of serious infections due to CRAb.^133^ In the primary outcome, the sulbactam-durlobactam group demonstrated a 28-day all-cause mortality rate of 19% (12 out of 63 patients) compared to 32% (20 out of 62 patients) in the colistin group, indicating a treatment difference of −13.2% (95% CI: −30.0 to 3.5). Furthermore, nephrotoxicity, a common issue with colistin, was significantly less frequent in the sulbactam-durlobactam group (13% versus 38%). Based on these robust clinical data, sulbactam-durlobactam is the preferred treatment in combination with a carbapenem, where it is available for clinical use. Accordingly, sulbactam-durlobactam was added to the most recent IDSA guidance for the treatment of resistant Gram-negative infections (Table 1),^130^ while the most recent ESCMID guidelines (2022) do not yet include sulbactam-durlobactam.^129^ Notably, while FDA granted fast-track, priority review, and generating antibiotic incentives now designations for this agent,^136^ as of April 2025, the EMA has not given regulatory approval for sulbactam-durlobactam. Though additional work is needed to understand the best combination strategies, we anticipate sulbactam-durlobactam to be the preferred backbone agent for available markets in future guidelines given currently available evidence and treatment options for CRAb.

**Table 2.** Phase III/IV clinical trials from 2014 to 2024 examining therapeutic options for CRAb

Trial	NCT	Study sites	Total sample size (n with CRAb)	Intervention	Comparator	Statistical design	Primary outcome	Key finding
ATTACK: Study to Evaluate the Efficacy and Safety of Intravenous Sulbactam-Durlobactam in the Treatment of Patients with Infections Caused by *Acinetobacter baumannii-calcoaceticus* Complex^133^	NCT03894046	59 sites, 16 countries	181 (181)	Sulbactam-durlobactam PLUS imipenem-cilastatin	Colistin PLUS Imipenem-cilastatin	Non-inferiority	28-d all-cause mortality	19% mortality in intervention group versus 32% in active comparator group Mortality difference of a 13.2% (95% confidence interval [CI]: −30.0 to 3.5) Non-inferiority margin met
CREDIBLE-CR Study of Cefiderocol or Best Available Therapy for the Treatment of Severe Infections Caused by Carbapenem-resistant Gram-negative Pathogens^134^	NCT02714595	95 sites, 16 countries	118^a^ (56)	Cefiderocol	Best available therapy (BAT, 1–3 agents, polymyxin or non-polymyxin based regimens)	Not specified	Primary outcome: Clinical cure at 7d after completion of therapy (pneumonia, sepsis, or bloodstream infection), microbiologic eradication (cUTI patients) Secondary: all-cause mortality at 14 and 28 days	Clinical cure: 53% (95% CI: 41, 63.8) versus 50% (95% CI: 33.4, 66.6) 28-day mortality 25% (95% CI: 16.7, 34.3) in cefiderocol arm versus 18% (95% CI: 8.8, 32) in BAT arm In patients with CRAb: 49% mortality with cefiderocol versus 18% with BAT Concerns about baseline imbalances in CRAb subgroup favoring more ill patients in cefiderocol group
Multicentre Open-label RCT to Compare Colistin Alone versus Colistin Plus Meropenem^132^	NCT01732250	6 sites	406 (312)	Colistin plus meropenem	Colistin monotherapy	Superiority	Clinical success at 14 days	79% clinical success in intervention arm; 73% in active comparator (risk difference −5.7%, 95% CI: −13.9 to 2.4; risk ratio 0.93, 95% CI: 0.83 to 1.03). Meropenem plus colistin not superior to colistin alone
Trial for the Treatment of Extensively Drug-Resistant Gram-negative Bacilli (OVERCOME)^131^	NCT01597973	19 sites, 7 countries	423^a^ (329)	Colistin plus meropenem	Colistin PLUS placebo	Superiority	28-day mortality Secondary outcomes: clinical failure, microbiological cure	28-day mortality of 43% versus 37%, no statistically significant difference (*P* = 0.17). No difference in secondary outcomes of clinical failure (65% versus 58%; difference of 6.8%, 95% CI: −3.1 to 16.6) and microbiologic cure (65% versus 60%; difference of 4.8%, 95% CI: −5.6 to 15.2).
Cefiderocol versus high-dose, extended-infusion meropenem for the treatment of Gram-negative nosocomial pneumonia (APEKS-NP)^135^	NCT03032380	119 sites	251 (47)	Cefiderocol plus linezolid	Meropenem PLUS linezolid	Non-inferiority	14-day mortality in MITT population	Among CRAb patients: 14-day mortality did not differ (19% with cefiderocol, 22% with meropenem; difference −3.0%, 95% CI: −24.8 to 18.8). Non-significant differences in rates of clinical cure and microbiological eradication among patients with *A. baumannii* in cefiderocol versus meropenem arms.

BAT, best alternative therapy; CRAb, carbapenem-resistant *Acinetobacter baumannii*; MITT, microbiologic modified intention to treat; cUTI, complicated urinary tract infection; RCT, randomized control trial.

^a^Microbiological modified intention-to-treat population.

Cefiderocol is a novel siderophore cephalosporin with *in vitro* activity against a wide range of multidrug-resistant Gram-negative bacteria, including CRAb. It was approved by the FDA in 2019 and EMA in 2020 based on the results of a series of pivotal clinical trials targeting UTI, HABP/VABP, and carbapenem-resistant organisms.^134,135,137^ The latter trial (CREDIBLE-CR Trial) was a Phase 3 randomized, open-label study evaluating the efficacy and safety of cefiderocol compared to the best available therapy (BAT) in adults with serious infections caused by carbapenem-resistant Gram-negative bacteria.^134^ Cefiderocol was well tolerated, and the clinical cure or microbiological eradication rates were comparable for nosocomial pneumonia, BSI/sepsis, and complicated UTI. However, there was excess mortality in the cefiderocol group (34%) compared to the BAT group (18%), particularly among patients with *Acinetobacter* spp. infections, raising concerns about its safety in these patients.^134^ Since its approval, real-world data, in which cefiderocol is typically used in combination with a second active agent, have reported comparable or improved clinical outcomes over comparators for CRAb infection.^138,139^ The potential utility of cefiderocol in the treatment of CRAb infection is therefore yet to be fully defined.

Novel agents and treatment strategies to address CRAb are an area of ongoing research focus. One such novel agent target inhibits the LptB2FGC complex to block the transport of lipopolysaccharide from the inner to the outer bacterial membrane.^140^ The clinical candidate, zosurabalpin, a macrocyclic peptide antibiotic, overcomes carbapenem resistance mechanisms *in vitro* and in mouse models and is currently being developed.^141^ Its actions are specific to *A. baumannii*, which gives hope for a potential targeted agent with a novel mechanism of action. Beyond antimicrobial development, alternative treatment strategies are being pursued. Phage therapy has been considered and holds promise as a future multimodal treatment approach; however, phage trials are difficult to conduct, and most data are from case reports.^142^ Finally, monoclonal antibodies, including MAb C8 and 65 are being considered as novel treatment approaches alongside antibiotics. Their mechanism of action is through targeting the capsule of *A. baumannii*, enhancing macrophage opsonophagocytosis, and evading sepsis; preclinical data show promise alone and in combination with antibiotics; however, clinical data in humans are needed.^143,144^

### Conclusion

CRAb is a challenging pathogen affecting the most vulnerable, critically ill patients and contributing substantial healthcare utilization and costs and significant mortality. The global landscape continues to change and requires ongoing epidemiologic surveillance. Treatment is often difficult and requires multiple agents. There is a critical need to develop and broadly implement rapid diagnostic tests that support pathogen identification and genotypic/phenotypic resistance detection. Future research will provide needed insight on the real-world outcomes in the era of sulbactam-durlobactam and high-dose ampicillin-sulbactam-based combinations. Robust efforts to prevent transmission in hospital settings and promote antimicrobial stewardship remain critical to addressing this urgent global threat, especially in areas of the world where access to novel therapeutic agents is limited.

## Transparency declarations

AB reports grants paid to her institution by the National Institutes of Health (K23HD113839), support from the Biogen Foundation, research support from the Thrasher Fund, consultancy from Elion Therapeutics, and serves on a data safety monitoring committee member for Innoviva Speciality Therapeutics. YD reports grants paid to his institutions by the National Institutes of Health (R01AI104895), Japan Agency for Medical Research and Development, Japan Society for the Promotion of Science, and serving on advisory boards for GSK, Shionogi and Meiji Seika Pharma.
